# *Campylobacter jejuni* strains isolated in Brazil have important virulence related genes and survive to different stress conditions

**DOI:** 10.1007/s42770-026-01928-6

**Published:** 2026-04-01

**Authors:** Miliane Rodrigues Frazão, Amanda Aparecida Seribelli, Carolina Nogueira Gomes, Giovana do Nascimento Pereira, Guojie Cao, Marta Inês Cazentini Medeiros, Sheila da Silva Duque, Marc William Allard, Juliana Pfrimer Falcão

**Affiliations:** 1 Department of Clinical Analyses, Toxicology and Food Science, School of Pharmaceutical Sciences of Ribeirão Preto, University of São Paulo, FCFRP-USP, Avenida Do Café, S/N., Bloco S - Sala 41, Ribeirão Preto, SP Brazil; 2 Ribeirão Preto Medical School, University of São Paulo, FMRP-USP, Ribeirão Preto, SP Brazil; 3 Division of Microbiology, Office of Regulatory Science, Center for Food Safety and Applied Nutrition, U.S. Food and Drug Administration, College Park, MD USA; 4 Adolfo Lutz Institute of Ribeirão Preto, Ribeirão Preto, SP Brazil; 5 Oswaldo Cruz Foundation (Fiocruz), Rio de Janeiro, RJ Brazil

**Keywords:** *Campylobacter jejuni*, Pathogenic potential, Virulence related genes, Whole genome sequencing, Resistance to different stress conditions, Brazil

## Abstract

*Campylobacter jejuni* has been reported as the leading bacterial pathogen that causes foodborne gastroenteritis in humans in many countries. This study aimed to assess the survival of 46 *C. jejuni* strains isolated from food (14), humans (18), and animals (14) in Brazil from 1996–2016 under different stress conditions, evaluate their invasion and survival capacity in Caco-2 cells and U-937 macrophages and determine virulence using the *Galleria mellonella* model for 23 selected strains. Furthermore, this study aimed to analyze the frequency of virulence related genes using WGS data. All 46 *C. jejuni* studied survived to acid stress with survival rates ranging from 29 to 100%. All studied strains survived to incubation in BHI with a final concentration of 7.5% NaCl with survival rates ranging from 3 to 100%. All strains showed growth in the temperatures analyzed with survival rates ranging from 5 to 100% at 4ºC and from 50 to 100% at 37ºC. Only two strains survived after 1 h of incubation under oxidative stress. All strains studied showed the ability to invade Caco-2 cells and to survive in U-937 macrophages. Regarding virulence in *G. mellonella*, 64% of strains presented virulence equal to or greater than the reference strain. The majority of the virulence genes detected was related to different stress conditions, adhesion, motility, and invasion. In conclusion, the pathogenic potential of the *C. jejuni* strains studied was highlighted and the results obtained may suggest that more rigorous control measures of this pathogen in food, especially poultry products are needed.

**Supplementary Information:**

The online version contains supplementary material available at 10.1007/s42770-026-01928-6.

## Introduction

*Campylobacter jejuni* has been reported as the leading cause of bacterial foodborne gastroenteritis in many countries [1–3]. According to the US Centers for Disease Control and Prevention [4], *Campylobacter* spp. have been the most common cause of diarrhea in humans affecting more than 1.5 million people annually in the United States. In 32 European countries, campylobacteriosis was the most frequently reported bacterial pathogen causing gastrointestinal infections in humans, with approximately 220,000 cases and a rate of 59.7 per 100,000 population in 2019 [5].

*C. jejuni* is most commonly transmitted through the consumption of raw or undercooked poultry meat, but can also be spread through contaminated water, unpasteurized milk, cross-contamination with other foods, and contact with other animals such as poultry, cattle, pigs, and other domesticated animals [6, 7].

Brazil produced 14,833million tons of chicken meat and exported 5,139 million tons in 2023, maintaining its position as the world’s largest poultry exporter since 2004 and the third largest producer of chicken meat [8]. However, campylobacteriosis has been a neglected disease, and relatively few studies have characterized *Campylobacter* spp. isolated from chicken meat in Brazil [9–16].

Clinical symptoms of campylobacteriosis commonly present as an acute gastroenteritis characterized by inflammation, abdominal pain, fever, diarrhea, and vomiting, also can lead to serious medical sequelae, such as Guillain-Barré syndrome, an autoimmune disorder that affects the peripheral nervous system and may lead to temporary paralysis [3, 17].

When food contaminated with *Campylobacter* is ingested, the bacteria reaches the stomach, the intestinal tract, and the feces, facilitating its transmission to a new host [3, 6]. During colonization of the human host, *C. jejuni* is exposed to inorganic acid (H^+^) in the gastric fluid of the stomach followed by exposure to organic acids in the small intestine. Adaptations to environmental conditions such as hyperosmotic environments, changes in nutrient availability, temperature variations, and reactive oxygen compounds, among others are crucial for the survivability of *Campylobacter*. The ability to counteract these environmental stresses is fundamental for the bacterium's survival [18–20].

*Campylobacter* has various defense strategies to mitigate stressful effects, such as enzymes SodB, AhpC, and KatA involved in cell detoxification and in aerobic stress [21, 22], production of heat shock proteins DnaK, GrpE, HspR in response to heat or acid stress [21, 23], HtrA, CsrA, PerR, CcpA-2, and Fur involved in response to the oxidative stress [21, 24, 25], and HupB, ProS, RpoB, ArgF, and Asd closely linked in response to cold stress [21, 26], among others. So, these genetic mechanisms have enabled the bacteria to adapt to these adverse environmental conditions.

Thus, the aims of this study were to assess the survival of *Campylobacter jejuni* strains isolated from diverse sources in Brazil under different stress conditions, the capacity of invasion and survival in Caco-2 cells and U-397 macrophages, the virulence in *Galleria mellonella* and to evaluate the frequency of virulence genes related to those stresses conditions to provide additional information about the pathogenic potential of strains of this species isolated in Brazil which may lead to the choice of better control measures to avoid poultry meat contamination by *C. jejuni*.

## Material and methods

### Bacterial strains

A total of 46 *C. jejuni* strains isolated from different sources were studied. The isolates were obtained from humans (18 strains), animals (14 strains), and food (14 strains) in the states of São Paulo, Minas Gerais, Rio de Janeiro, and Rio Grande do Sul, located in the Southeast and South regions of Brazil, between 1996 and 2016. These strains were selected from the collections of the *Campylobacter* References Laboratories of the Oswaldo Cruz Institute of Rio de Janeiro (Fiocruz-RJ) and of the Adolfo Lutz Institute of Ribeirão Preto (IAL-RP) in Brazil. They were systematically chosen to represent isolates from sporadic cases from different clinical and non-clinical samples of the two collections of the reference laboratories mentioned above that occurred during different years. Table 1 summarizes the isolation data and accession numbers of the 46 *C. jejuni* strains analyzed in this study. All experiments described in this study were performed using the 46 *C. jejuni* strains listed in Table 1, except for the *Galleria mellonella* infection model, in which a subset of 23 strains was selected based on their phenotypic performance in the *in vitro* assays.

**Table 1:** Isolation data and accession numbers of the 46 *Campylobacter jejuni* strains studied isolated from humans (*n* = 18), animals (*n* = 14) and food (*n* = 14) in Brazil between 1996 and 2016

CFSAN *n*°	Isolate name	GenBank accession *n*°	Source	State	Year
CFSAN065338	CCAMP 487^#^	PHZV00000000	Human diarrheal faeces	RJ	1996
CFSAN065340	CCAMP 489^#^	PHZT00000000	Human diarrheal faeces	RJ	1997
CFSAN065342	CCAMP 497	PHZR00000000	Human diarrheal faeces	RJ	1998
CFSAN065344	CCAMP 506^#^	PHZP00000000	Human diarrheal faeces	RJ	2000
CFSAN065346	CCAMP 588	PHZN00000000	Human diarrheal faeces	RJ	2001
CFSAN065294	Cj 01	PIBJ00000000	Human diarrheal faeces	SP	2002
CFSAN065296	Cj 03	PIBH00000000	Human diarrheal faeces	SP	2003
CFSAN065299	Cj 07^#^	PIBE00000000	Human blood	SP	2003
CFSAN065348	CCAMP 601	PHZL00000000	Human diarrheal faeces	RJ	2003
CFSAN065360	CCAMP 699^#^	PHZA00000000	Human diarrheal faeces	RJ	2004
CFSAN065306	Cj 16	PIAY00000000	Human diarrheal faeces	SP	2005
CFSAN065309	Cj 19^#^	PIAV00000000	Human diarrheal faeces	SP	2006
CFSAN065315	Cj 25^#^	PIAQ00000000	Human diarrheal faeces	SP	2007
CFSAN065317	Cj 27	PIAP00000000	Human diarrheal faeces	SP	2008
CFSAN065320	Cj 30	PIAN00000000	Human diarrheal faeces	SP	2009
CFSAN065404	CCAMP 1491^#^	PHXJ00000000	Human blood	RJ	2011
CFSAN065406	CCAMP 1497	PHXH00000000	Human diarrheal faeces	RJ	2014
CFSAN065324	Cj 34^#^	PIAJ00000000	Human diarrheal faeces	SP	2015
CFSAN065364	CCAMP 770^#^	PHYW00000000	Monkey non-diarrheal faeces	RJ	1996
CFSAN065366	CCAMP 828	PHYU00000000	Monkey non-diarrheal faeces	RJ	1997
CFSAN065370	CCAMP 991	PHYQ00000000	Monkey non-diarrheal faeces	RJ	1999
CFSAN065355	CCAMP 685	PHZE00000000	Monkey non-diarrheal faeces	RJ	2000
CFSAN065327	CCAMP 162^#^	PIAG00000000	Monkey non-diarrheal faeces	RJ	2003
CFSAN065332	CCAMP 473^#^	PIAB00000000	Chicken non-diarrheal faeces	MG	2004
CFSAN065335	CCAMP 479^#^	PHZY00000000	Chicken non-diarrheal faeces	MG	2004
CFSAN065337	CCAMP 481^#^	PHZW00000000	Chicken non-diarrheal faeces	MG	2004
CFSAN065351	CCAMP 672^#^	PHZI00000000	Chicken non-diarrheal faeces	RJ	2006
CFSAN065399	CCAMP 1080	PHXO00000000	Monkey non-diarrheal faeces	RJ	2009
CFSAN065405	CCAMP 1493	PHXI00000000	Monkey non-diarrheal faeces	RJ	2013
CFSAN065412	CCAMP 1538	PHXC00000000	Chicken non-diarrheal faeces	RS	2015
CFSAN065413	CCAMP 1555	PHXB00000000	Chicken non-diarrheal faeces	RJ	2015
CFSAN065414	CCAMP 1574^#^	PHXA00000000	Chicken non-diarrheal faeces	RJ	2016
CFSAN065371	CCAMP 1013	PHYP00000000	Chicken pieces	MG	2008
CFSAN065373	CCAMP 1015	PHYN00000000	Chicken pieces	MG	2008
CFSAN065376	CCAMP 1019	PHYK00000000	Chicken pieces	MG	2008
CFSAN065378	CCAMP 1021^#^	PHYI00000000	Chicken pieces	MG	2008
CFSAN065381	CCAMP 1025	PHYF00000000	Chicken pieces	MG	2008
CFSAN065383	CCAMP 1039	PHYD00000000	Chicken pieces	MG	2008
CFSAN065385	CCAMP 1048^#^	PHYB00000000	Chicken pieces	MG	2008
CFSAN065388	CCAMP 1052^#^	PHXY00000000	Chicken pieces	MG	2009
CFSAN065390	CCAMP 1054	PHXW00000000	Chicken pieces	MG	2009
CFSAN065394	CCAMP 1058^#^	PHXS00000000	Chicken pieces	MG	2009
CFSAN065397	CCAMP 1061^#^	PHXP00000000	Chicken pieces	MG	2009
CFSAN065407	CCAMP 1518	PHXG00000000	Chicken carcass	RS	2015
CFSAN065409	CCAMP 1520^#^	PHXE00000000	Chicken carcass	RS	2015
CFSAN065411	CCAMP 1523^#^	PITS00000000	Chicken carcass	RS	2015

*MG* Minas Gerais, *SP* São Paulo, *RJ* Rio de Janeiro, *RS* Rio Grande do Sul

^#^virulence analysis in *G. mellonella*

### Genus and species confirmation

The genomic DNAs of the strains listed in Table 1 were extracted according to [27], with a few modifications. Specifically, the strains were cultured at 42 ºC on BBL Columbia Agar Base (Becton Dickinson), supplemented with charcoal (Neon) and FBP [(0.5% ferrous sulfate (Labsynth), 0.5% sodium pyruvate (Vetec) and 0.5% sodium metabisulphite (Labsynth) diluted in sterile water] under microaerobic conditions (10% CO_2_, 5% O_2_ and 85% N_2_) for 18–24 h.

After incubation, colonies displaying typical *C. jejuni* morphology, characterized by small size (approximately 1 to 2 mm in diameter), smooth, round and a translucent to grayish appearance, were selected for genomic DNA extraction. The growth of the strains was placed directly in Solution 1 (20% sucrose, 50 mM Tris/HCl, pH 8.0, 50 mM EDTA) of the extraction protocol.

The quality of the DNAs was checked using NanoDrop 1000 (Thermo Scientific, Rockford, IL), and the concentrations were determined by Qubit double-stranded DNA BR assay kit and Qubit fluorometer (Life Technologies, Grand Island, NY) according to each manufacturer's instructions. The molecular confirmation of the genus and species was done using the specific regions of the 16S *rRNA* (confirmation of the genus *Campylobacter*), *ceuE* (confirmation of *C. coli*), and *mapA* (confirmation of *C. jejuni*) genes as described by [28].

### Stress condition assays

#### Tolerance to temperature variations

The 46 *C. jejuni* strains studied were grown at 42 ºC overnight as described in item 2.2. After incubation, the bacterial growth was collected from the plate, inoculated in 9 mL of BHI broth, and incubated at 42 ºC under a microaerobic atmosphere for 16 h to obtain stationary phase cells. Bacterial growth was adjusted to an OD_600_ = 0.1 which was previously shown to correspond to approximately 8 log_10_ CFU/ml [19] and subsequently used as an inoculum to analyze the effects of low temperature storage at 4 ºC for 24 h and incubation at 37 ºC for 24 h under microaerophilic conditions. Aliquots were taken at 30 min and 24 h and the strains grown at 42 ºC were used as control in the analyses. The CFU/mL were determined by serial dilutions and plating on Muller Hinton supplemented with 5% sheep's blood. Experiments were conducted in three independent replicates.

#### Survivability to acid and oxidative stress

In the early stationary phase reached after 16 h of incubation, the *C. jejuni* cultured in BHI had their optical density adjusted to an OD_600_ = 0.1 and 1 mL was centrifuged at 8000 × g for 5 min. To perform the acid stress, pellets were resuspended in 1 mL of acid Brain Heart Infusion (BHI) broth (pH = 4.5) and incubated at 42 ºC under microaerophilic conditions as described by [20] with modifications. Acid broth was prepared by adding hydrochloric acid (HCl) directly to the BHI broth and the pH was measured using a pH meter. Aliquots were taken at 10 min, 1 h, and 2 h after exposure to stress and strains resuspended in BHI pH = 7 were used as a control in the analyses. The CFU/mL was determined by serial dilutions and plating on Muller Hinton supplemented with 5% sheep's blood. Experiments were conducted in three independent replicates.

To conduct the oxidative stress analysis, strains were first incubated at 42 ºC under aerophilic conditions for 24 h. The strains that presented growth were subjected to oxidative stress. After this, new cultures of *C. jejuni* were performed, and in the early stationary phase, these cultures had their optical density adjusted to OD_600_ = 0.1 and were centrifuged as described above. Pellets were resuspended in 10 mL of saline solution (0.8%) supplemented with H_2_O_2_ [15 mM]. Aliquots were taken at 10 min and 1 h after exposure to oxidative stress as described by [18], with some modifications, and strains resuspended in saline solution without H_2_O_2_ supplementation were used as control in the analyses. The CFU/mL was determined by serial dilutions and plating on Muller Hinton supplemented with 5% sheep's blood. Experiments were conducted in three independent replicates.

#### Survivability in 7.5% of NaCl

The inoculum was obtained according to item 2.3. After adjusting the optical density to an OD_600_ = 0.1, 1 mL of stationary phase cells was centrifuged at 8000 × g for 5 min and subsequently resuspended in 1 mL of Brain Heart Infusion (BHI) broth to which sodium chloride was added to reach a final concentration of 7.5% NaCl (wt/vol), as described by [20] with few modifications. The only modification was the NaCl concentration, which was enhanced from 3% to 7.5%. All strains studied were incubated for 2 h at 42 ºC under microaerophilic conditions. Aliquots were taken at 10 min, 1 h, and 2 h after exposure to stress and strains resuspended in BHI without NaCl addition were used as a control in the analyses. CFU/mL were determined by serial dilutions and plating on Muller Hinton supplemented with 5% sheep's blood and the survivability was assessed by determining the survival percentage of viable and culturable *C. jejuni* cells after exposure to salt stress. Experiments were conducted in three independent replicates.

### *In vitro* phenotypic assays

#### Invasion assay in Caco-2 epithelial cells and survival assay in U937 human macrophages

These assays were performed for all 46 *C. jejuni* strains (Table 1) and the reference strain *C. jejuni* ATCC 33291. The invasion assay in Caco-2 epithelial cells was used to evaluate the ability of the strains to invade intestinal epithelial cells, mimicking their interaction with the human intestinal epithelium, whereas the survival assay in U937 human macrophages was used to assess the ability of the strains to survive within immune cells, reflecting their potential to evade host immune responses.

Initially, the Caco-2 epithelial cells were cultured in DMEM medium (Dulbecco’s Modified Eagle Medium – Gibco - low glucose) supplemented with 10% fetal bovine serum (Life Technologies) and antibiotic in 5% CO_2_ at 37 °C. Subsequently, 1 × 10^5^ cells were added to each well of a 12-well microplate. The assay was performed after 12 days of incubation until the cells were polarized and differentiated [29, 30].

The monocytes were cultured in suspension in RPMI medium (1640 – powder - Gibco) supplemented with 10% fetal bovine serum (Life Technologies) and antibiotic in 5% CO^2^ at 37 °C. Thereafter, 1 × 10^5^ cells were added to each well of a 24-well microplate. For the differentiation of monocytes into macrophages, 1 μl of phorbol 12-myristate-13-acetate (PMA) (Sigma-Aldrich) was used in 50 mL of the RPMI medium (50 ng/mL) and maintained in 5% CO^2^ at 37 °C for 48 h. All the *C. jejuni* strains were opsonized with 20% mouse serum (Sigma-Aldrich) at 37 °C for 15 min after three washes with PBS (centrifuged at 12000 × rpm for 1 min).

The invasion assay in Caco-2 epithelial cells was performed after 90 min of bacteria–cell interaction, while the survival assay in U937 human macrophages was performed after 30 min of interaction. In both assays, the multiplicity of infection (MOI) was 100:1. After the interaction period, extracellular bacteria were eliminated by treatment with gentamicin (30 µg/mL) for 90 min. The cells were then washed with PBS and incubated in antibiotic-free medium for 3 h to allow intracellular survival. Subsequently, the cells were washed again with PBS and lysed with 1% Triton X-100 for 5 min to release intracellular bacteria. The CFU/mL was determined by serial dilutions and plating on Mueller–Hinton agar supplemented with 5% sheep blood with incubation for 18–24 h at 42 ºC under microaerophilic conditions. The experiments were carried out in biological triplicate and in all plates, there was a negative control with only cells in the wells.

### *In vivo* infection model

#### Virulence analysis in *Galleria mellonella*

The analysis was performed according to [31]. The larvae were maintained at 28 °C in the dark in glass containers (30 cm height - 20 cm wide - 2 L capacity) with appropriate oxygen and access to food until reaching the sixth instar, whose weight is between 200 and 250 mg. After complete development, the larvae were deprived of food and separated into groups of 10 units in glass Petri dishes for each bacterial isolate and controls.

Based on the results of the *in vitro* phenotypic assays described above, 23 strains showing survival rates greater than or equal to those of the reference strain ATCC 33291 were selected for evaluation in the *Galleria mellonella* infection model.

For the infection assay, *C. jejuni* strains were grown in BHI broth and harvested at the early stationary phase (16 h of incubation). Bacterial suspensions were adjusted to an OD₆₀₀ of 0.1, and 1 mL aliquots were centrifuged at 8,000 × g for 5 min. The pellets were washed and resuspended in 1 mL of PBS and then a Hamilton micro-syringe (model 7000.5KH of 10 μL) was used for artificial inoculation of *G. mellonella* larvae into the center of the last right pro-leg with 10 μL of the suspension for each of the strains studied and for the positive control infected with *C. jejuni* ATCC 33291. The negative control was inoculated with PBS. After inoculation, the larvae were incubated at 37 °C and deprived of food and direct light. During the 10-day experimental period, larvae were examined every 24 h and pre-pupae were removed to delay metamorphosis. Larval survival was recorded daily, and larvae were considered dead when they showed no response to touch. The percentage of larval mortality was used to determine the virulence of each strain.

### Genome sequencing, assembly, and annotation

All isolates were prepared using the Nextera Sample Preparation Kit (Illumina, San Diego, CA) and then sequenced on a MiSeq or a NextSeq (Illumina) using a 2 × 250-bp or a 2 × 150-bp paired-end MiSeq or NextSeq reagent kit, respectively. *De novo* assemblies were generated from all raw sequence data. The Illumina reads were assembled with CLC Genomics Workbench version 10.0.1 (CLC Bio, Aarhus, Denmark) [32]. The contigs for each isolate (draft genome) were annotated using NCBI's Prokaryotic Genomes Automatic Annotation Pipeline (PGAAP) (Klimke et al., 2009).

### Nucleotide sequence accession numbers

WGS assemblies of 46 *Campylobacter jejuni* strains of this study were submitted to the National Center for Biotechnology Information and the GenBank accession numbers of each strain are listed in Table 1.

### Virulence related genes characterization

The virulence genes in all *C. jejuni* strains were identified using ABRicate software (available at https://github.com/tseemann/abricate/), which performs a screening for virulence genes in assemblies submitted based on Virulence Factors Database (VFDB).

In additional, we searched the presence of the 20 virulence-related genes (*katA*, *perR*, *fur*, *csrA*, *sodB*, *ahpC*, *ccpA-2*, *tpx*, *htrA*, *fdxA*, *dcuA*, *grpE*, *hspR*, *dnaK*, *spoT*, *argF*, *asd*, *proS*, *hupB* and *rpoB*) in the WGS assemblies of all strains studied with BLASTn (https://blast.ncbi.nlm.nih.gov/Blast.cgi) and the sequence of each gene was assessed using NCBI (https://ncbi.nlm.nih.gov) [33].

### Statistical analyses

The comparisons between the means of the virulence tests and the cells assays were performed using Student's t-test for two means and two-way analysis of variance (ANOVA) with post hoc Tukey test for more than two means in the Minitab® statistical software (version 18.1). For all analyses, the level of significance was α = 5%.

The graphics of the virulence assay in *Galleria mellonella* were performed using the Prism5 program for Windows of the GraphPad® software (version 5.01). For all analyses, the level of significance was α = 5%.

## Results

### Genus and species confirmation

All strains analyzed in this study were confirmed to belong to the species *C. jejuni* by amplification of the 16S rRNA and *mapA* genes.

### Stress condition assays

#### Tolerance to temperature variations

**Fig. 1:**
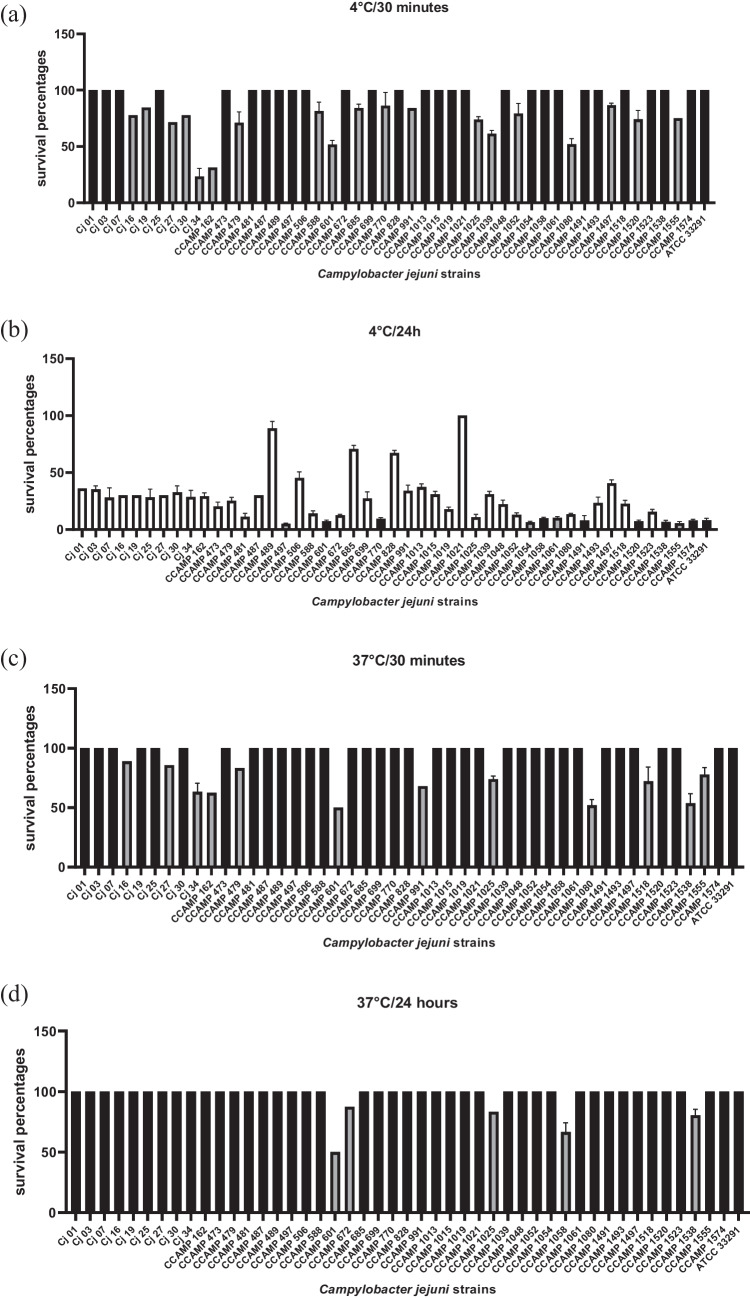
Survival of 46 *Campylobacter jejuni* strains and the control (*Campylobacter jejuni* ATCC 33291) at 4 °C after 30 min (**a**) and 24 h (**b**) and at 37 °C after 30 min (**c**) and 24 h (**d**) of incubation under microaerophilic conditions. Results expressed as % of surviving cells. White bars: strains that survived more than reference strain ATCC 33291 (*p* < 0,05). Black bars: strains that survived equal than reference strain ATCC 33291. Gray bars: strains that survived less than reference strain ATCC 33291(*p* < 0,05). The error bars represent standard deviation of biological triplicate

All strains studied, including the reference strain *C. jejuni* ATCC 33291, showed growth in the two temperatures analyzed. The survivability of the studied strains was compared to the reference strain *C. jejuni* ATCC 33291 using Student’s t-test. After 30 min of incubation at 4 ºC, 27 (59%) strains survived equal and 19 (41%) strains survived less than the reference strain (Fig. 1a). In this temperature after 24 h of incubation, 35 (76%) strains survived more than the reference strain, 7 (15%) strains survived equal and 4 (9%) strains survived less) than the *C. jejuni* ATCC 33291 (Fig. 1b).

After 30 min of incubation at 37 ºC, 34 (74%) strains survived equal and 12 (26%) strains survived less than the reference strain (Fig. 1c). In this temperature after 24 h of incubation, 41 (89%) strains survived equal and 5 (11%) strains survived less than the *C. jejuni* ATCC 33291 (Fig. 1d). This analysis did not reveal any significant correlation between the origin of the *C. jejuni* strains and their tolerance to the temperature conditions evaluated.

#### Survivability to acid and oxidative stress

**Fig. 2:**
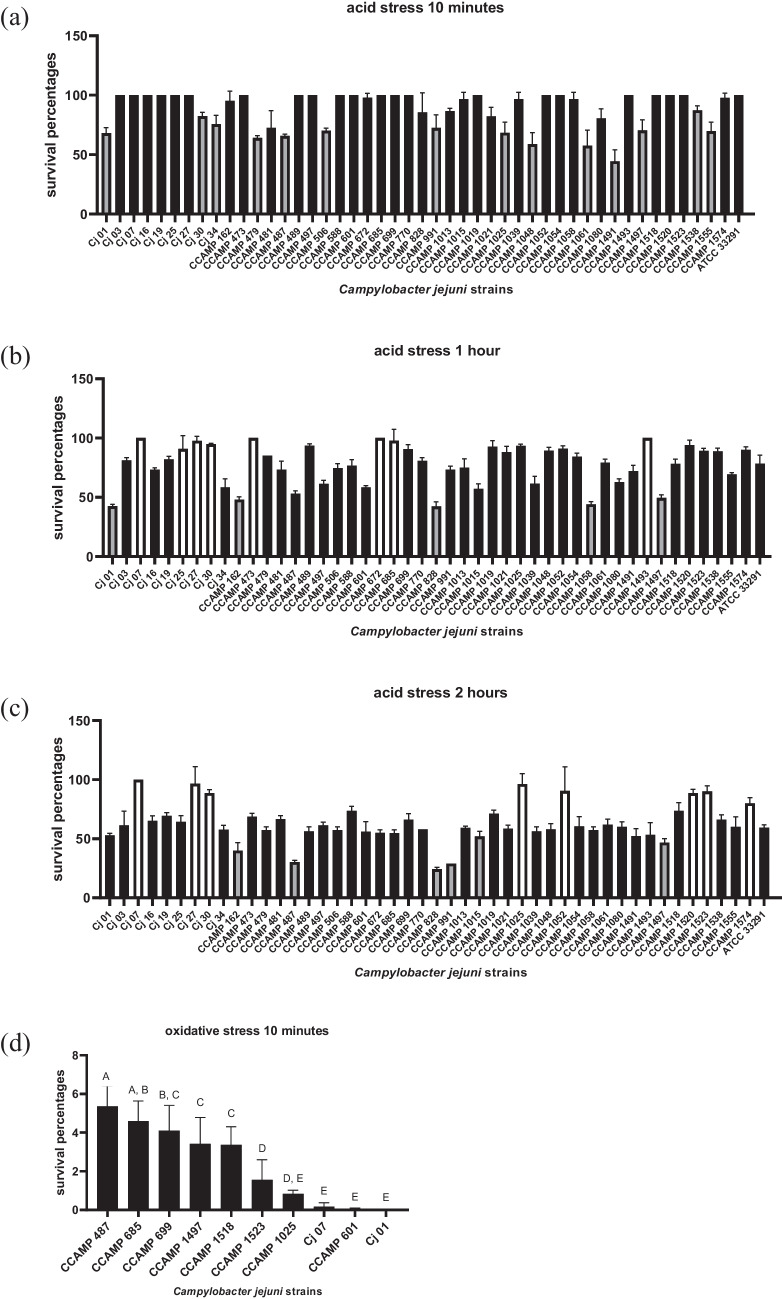
Survival of *Campylobacter jejuni* strains under stress conditions. Survival of 46 *C. jejuni* strains and the control strain (*C. jejuni* ATCC 33291) to acid stress at 42 °C under microaerophilic conditions after 10 min (**a**), 1 h (**b**), and 2 h (**c**) of incubation. Survival of 10 *C. jejuni* strains to oxidative stress at 42 °C under microaerophilic conditions after 10 min (**d**). Error bars represent the standard deviation of biological triplicates. (**a**, **b**, **c**) White bars: strains that survived more than reference strain ATCC 33291 (*p* < 0,05). Black bars: strains that survived equal than reference strain ATCC 33291. Gray bars: strains that survived less than reference strain ATCC 33291(*p* < 0,05). The error bars represent standard deviation of biological triplicate

All strains studied remained viable at various levels compared to *C. jejuni* ATCC 33291 after two hours of incubation in BHI pH = 4.5. From the statistical analysis of the Student’s t-test, after 10 min of incubation, 32 (70%) isolates that survived equal and 14 (30%) isolates that survived less than the *C. jejuni* ATCC 33291 (Fig. 2a). After 1 h of incubation, 8 (17%) isolates that survived more, 33 (72%) equal and 5 (11%) less (11%) than the *C. jejuni* ATCC 33291 (Fig. 2b). After 2 h of incubation, 8 (17%) strains survived more, 32 (70%) strains survived equal and 6 (13%) six strains survived less than the *C. jejuni* ATCC 33291 (Fig. 2c).

Sixteen (35%) strains studied (Cj 01, Cj 03, Cj 07, Cj 25, CCAMP 479, CCAMP 487, CCAMP 588, CCAMP 601, CCAMP 685, CCAMP 699, CCAMP 991, CCAMP 1013, CCAMP 1025, CCAMP 1497, CCAMP 1518 and CCAMP 1523) exhibited growth after incubation at 42 ºC under aerophilic conditions for 24 h and were subjected to oxidative stress. Ten of 16 strains subjected to oxidative stress survived after 10 min of incubation being 6 strains isolated from humans (Cj 01, Cj 07, CCAMP 487, CCAMP 601, CCAMP 699, and CCAMP1497), 3 strains isolated from food (CCAMP 1025, CCAMP 1518 e CCAMP 1523) and 1 strain isolated from animal (CCAMP 685) (Fig. 2d). For this essay no survival was observed to the reference strain *C. jejuni* ATCC 33291 and the bidirectional analysis of variance (ANOVA) categorized the 10 strains that survived after 10 min of incubation in groups from A to E (Fig. S1). Two strains (CCAMP 1025 and CCAMP 1523) isolated from food survived after 1 h of incubation. The survival percentages of CCAMP 1025 and CCAMP 1523 strains after 1 h of incubation were 5% e 0,4% respectively and according to Student’s test, a statistically significant difference was found (p-value < 0.05) between these survival percentages. This analysis did not reveal any significant correlation between the origin of the *C. jejuni* strains and their survivability under acid and oxidative stress conditions.

#### Survivability in 7.5% of NaCl

**Fig. 3:**
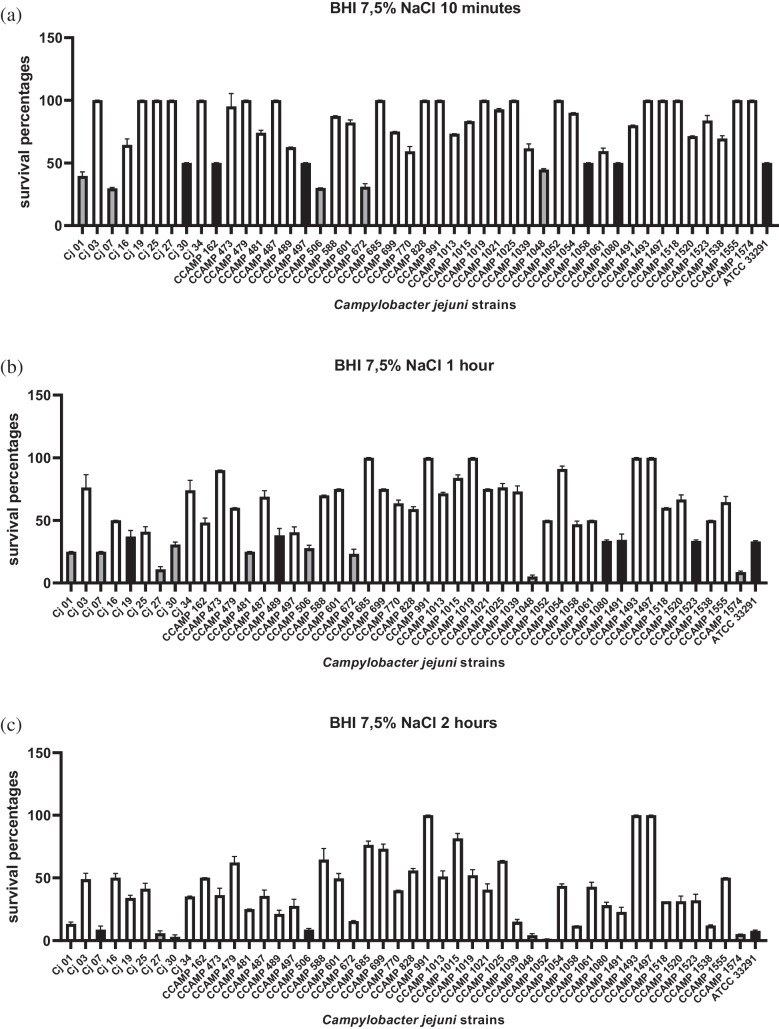
Survival of 46 *Campylobacter jejuni* strains and the control (*Campylobacter jejuni* ATCC 33291) in 7.5% of NaCl at 42 °C under microaerophilic conditions after 10 min (**a**), 1 h (**b**) and 2 h (**c**) of incubation. Results expressed as % of surviving cells. White bars: strains that survived more than reference strain ATCC 33291 (*p* < 0,05). Black bars: strains that survived equal than reference strain ATCC 33291. Gray bars: strains that survived less than reference strain ATCC 33291(*p* < 0,05). The error bars represent standard deviation of biological triplicate

All strains studied remained viable at various levels compared to *C. jejuni* ATCC 33291 after two hours of incubations in BHI with a final concentration of 7.5% NaCl (wt/vol). From the statistical analysis of the Student’s t-test, after 10 min of incubation, 36 (78%) isolates survived more, 5 (11%) isolates equal and 5 (11%) less than the *C. jejuni* ATCC 33291 (Fig. 3a). After 1 h of incubation, 32 (69%) strains survived more, 5 (11%) strains survived equal and 9 (20%) strains survived less than the *C. jejuni* ATCC 33291 (Fig. 3b). After 2 h of incubation, 39 (85%) strains survived more, 2 (4%) strains survived equal and 5 (11%) strains survived less (than the *C. jejuni* ATCC 33291 (Fig. 3c). This analysis did not reveal any significant correlation between the origin of the *C. jejuni* strains and their survivability in 7.5% of NaCl stress conditions.

### *In vitro* phenotypic assays

#### Caco-2 epithelial cells invasion assay

**Fig. 4:**
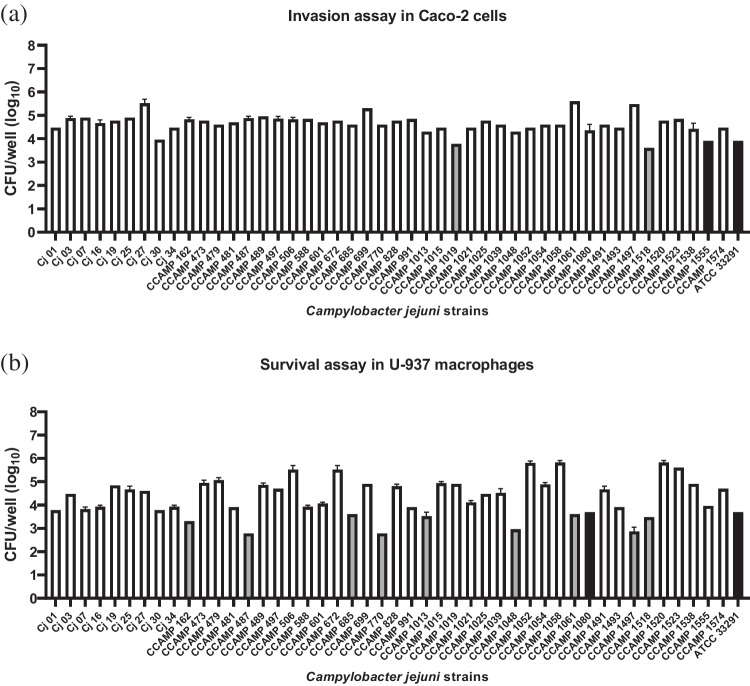
Invasion and survival assays of *Campylobacter jejuni* strains. Invasion assay in Caco-2 epithelial cells (**a**) and survival assay in U-937 human macrophages (**b**) for 46 *C. jejuni* strains isolated from different sources. Error bars represent the standard deviation of biological triplicates. White bars: strains that survived more than reference strain ATCC 33291 (*p* < 0,05). Black bars: strains that survived equal than reference strain ATCC 33291. Gray bars: strains that survived less than reference strain ATCC 33291(*p* < 0,05). The error bars represent standard deviation of biological triplicate

The invasion assay in Caco-2 cells demonstrated that all 46 *C. jejuni* strains studied had the capacity to invade these cells. Considering all strains studied, invasion in Caco-2 cells ranged from 1 × 10^4^ to 1 × 10^6^ CFU/mL. The invasiveness of the studied strains was compared to the ATCC 33291 reference strain using the Student's t-test and 43 (93%) strains invaded at higher levels than the ATCC 33291 reference strain, two strains invaded at lower levels than the reference strain, and one strain invaded the same as the reference strain (Fig. 4a). No significant association was identified between the origin of the *C. jejuni* strains and their invasion capacity in Caco-2 epithelial cells.

#### Survival assay in U937 human macrophages

The survival assay in U937 human macrophages demonstrated that all 46 *C. jejuni* strains studied had the capacity to survive in these cells at various levels compared to the ATCC 33291 reference strain. Considering all strains studied, survival in U937 human macrophages ranged from 1 × 10^3^ to 1 × 10^6^ CFU/mL. From the statistical analysis of Student's t-test, three subgroups were formed comprising: 36 (78%) isolates that survived more, 1 (2%) equal and 9 (20%) less than the *C. jejuni* ATCC 33291 reference strain (Fig. 4b). The survival of the *C. jejuni* strains in U937 human macrophages did not show any significant relationship with the origin of the isolates.

### *In vivo* infection model

#### Virulence analysis in *Galleria mellonella*

The results of the 23 *C. jejuni* strains (marked with # in Table 1), being nine isolated from humans, seven isolated from food, and seven isolated from animals, are presented below by source of isolation.

**Fig. 5:**
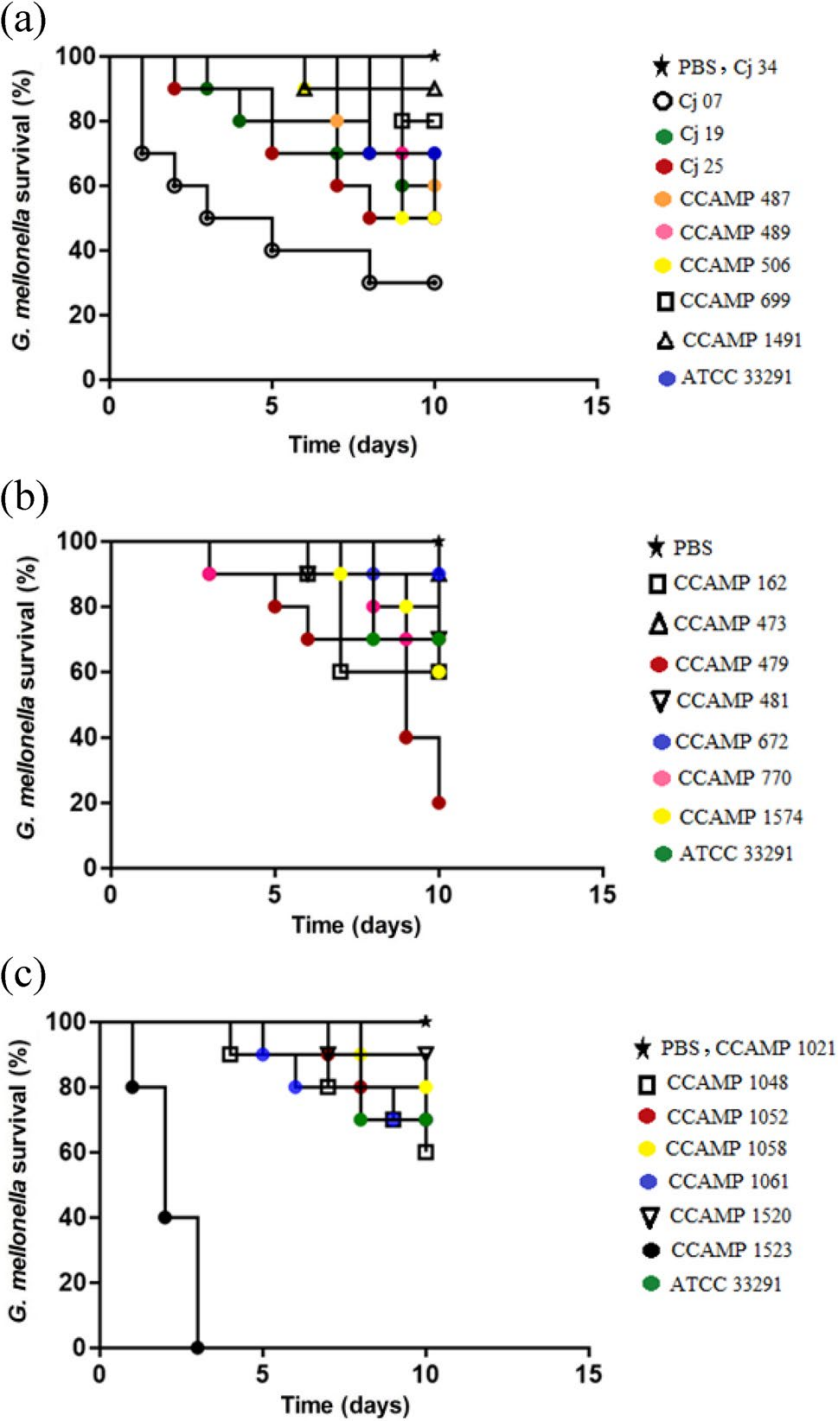
Survival percentages of *Galleria mellonella* larvae infected with *C. jejuni* strains isolated from humans (**a**), from animal (**b**) and from food (**c**) after ten days

Initially, the *C. jejuni* ATCC 33291 control strain showed intermediate virulence, killing 30% of the larvae. For the strains isolated from humans, the Cj 07 strain was the most virulent, killing 70% of the larvae. The Cj 19, Cj 25, CCAMP 487, CCAMP 489 and CCAMP 506 strains killed between 30 and 50% of the larvae, forming the group of intermediate virulence strains. The CCAMP 699 and CCAMP 1491 strains formed the group of low virulent strains, killing 10% to 20% of the larvae. The Cj 34 strain and the negative control PBS were avirulent and did not kill any larvae (Fig. 5a).

For animal isolates, the CCAMP 479 strain killed 80% of the larvae being the most virulent strain. The intermediate virulence group killed between 30–40% of the larvae, comprised of CCAMP 162, CCAMP 481, CCAMP 770, and CCAMP 1574 strains. The CCAMP 473 and CCAMP 672 formed the group of low virulence killing between 10–20% of the larvae (Fig. 5b).

For the strains isolated from food, the CCAMP 1523 strain was the most virulent, killing 100% of the larvae. The CCAMP 1048 and CCAMP 1061 strains killed between 30 and 40% of the larvae, forming the group of intermediate virulence strains. The CCAMP 1052, CCAMP 1058 and CCAMP 1520 strains formed the group of low virulent strains, killing 10% to 20% of the larvae. The CCAMP 1021 strain was avirulent and did not kill any larvae (Fig. 5c). The virulence patterns observed in the *G. mellonella* infection model were not associated with the origin of the *C. jejuni* strains.

### Virulence related genes frequencies

Genetic characterization focused on searching virulence-related genes using the Virulence Factors Database (VFDB) by ABRicate software detected 139 genes related to colonization, adhesion, motility, invasion, toxin production, chemotaxis, metabolism, and type 6 secretion system (T6SS). The coverage rate varied between 4 and 100% and the identity rate varied between 75 and 100%. Among the 139 genes, 84 were common to all strains, and the total number of genes per strain ranged from 96 to 119 (Table 2).

**Table 2:** Proportion of virulence genes found in the 46 *Campylobacter jejuni* strains studied

Genes	Quantity (%)	Coverage (%)	Identity (%)
Colonization			
*glf*	4/46 (9%)	98–100	100
*acfB*	5/46 (11%)	6	83
Adhesion			
* cadF*	46/46 (100%)	100	99–100
* Cj1135*	34/46 (74%)	34–100	85–99
* Cj1136*	30/46 (65%)	18–100	76–100
* Cj1137c*	6/46 (13%)	30–100	100
* Cj1138*	21/46 (46%)	22–100	77–100
* Cj1416c*	43/46 (93%)	100	98–100
* Cj1417c*	43/46 (93%)	100	97–100
* Cj1419c*	44/46 (96%)	99–100	83–100
* Cj1420c*	44/46 (96%)	99–100	83–100
*Cj1421c*	38/46 (83%)	4–61%	81–100
*Cj1422c*	7/46 (15%)	8–100	82–100
*Cj1426c*	4/46 (9%)	100	98–100
*Cj1427c*	13/46 (28%)	35–100	95–100
*Cj1432c*	4/46 (9%)	100	100
*Cj1434c*	4/46 (9%)	28–63	100
*Cj1435c*	4/46 (9%)	100	100
*Cj1436c*	4/46 (9%)	100	100
*Cj1437c*	4/46 (9%)	100	100
*Cj1438c*	4/46 (9%)	62–79	100
*Cj1440c*	18/46 (39%)	17–100	75–100
*cstIII*	5/46 (11%)	100	100
*gmhA*	46/46 (100%)	98–100	93–98
*gmhA2*	39/46 (85%)	98–100	92–100
*gmhB*	46/46 (100%)	23–100	93–100
*hddA*	39/46 (85%)	100	93–100
*hddC*	39/46 (85%)	96–100	89–100
*hldD*	46/46 (100%)	100	96–100
*hldE*	46/46 (100%)	60–100	93–100
*htrB*	46/46 (100%)	98–100	96–100
*jlpA*	46/46 (100%)	48–100	97–100
*kpsC*	46/46 (100%)	53–100	94–100
*kpsD*	46/46 (100%)	100	96–100
*kpsE*	46/46 (100%)	98–100	81–100
*kpsF*	46/46 (100%)	99–100	93–100
*kpsM*	46/46 (100%)	100	80–100
*kpsS*	46/46 (100%)	98–100	98–100
*kpsT*	46/46 (100%)	99–100	89–100
*neuA1*	36/46 (78%)	25–100	79–100
*neuB1*	26/46 (57%)	46–100	75–100
*neuC1*	(25/46) 54%	85–100	77–100
*pebA*	46/46 (100%)	100	98–100
*porA*	46/46 (100%)	97–100	78–98
*rfbC*	37/46 (80%)	48–100	77–100
*waaC*	46/46 (100%)	13–100	96–100
*waaF*	46/46 (100%)	96–100	94–100
*waaV*	46/46 (100%)	95–100	90–100
*wlaN*	24/46 (52%)	13–100	75–100
*tcpI*	46/46 (100%)	6	84–86
*pefA*	1/46 (2%)	46	98
*eptC*	46/46 (100%)	100	98–100
*ratB*	1/46 (2%)	4	100
*fcl*	15/46 (33%)	98–100	75–100
*kfiD*	4/46 (9%)	100	100
*galU*	1/46 (2%)	26	76
Motility/Invasion			
*flaA*	46/46 (100%)	5–100	82–100
*flaB*	46/46 (100%)	9–100	77–100
*flaC*	46/46 (100%)	100	94–100
*flaD*	46/46 (100%)	100	96–100
*flaG*	46/46 (100%)	100	97–100
*flgA*	46/46 (100%)	100	99–100
*flgB*	46/46 (100%)	100	97–100
*flgC*	46/46 (100%)	100	99–100
*flgD*	46/46 (100%)	99–100	97–100
*flgE*	46/46 (100%)	42–100	82–100
*flgF*	46/46 (100%)	100	99–100
*flgG*	46/46 (100%)	100	99–100
*flgH*	46/46 (100%)	100	98–100
*flgI*	46/46 (100%)	100	98–100
*flgJ*	46/46 (100%)	100	96–100
*flgK*	46/46 (100%)	100	92–100
*flgM*	46/46 (100%)	100	98–100
*flgP*	46/46 (100%)	100	100
*flgQ*	46/46 (100%)	100	100
*flgR*	46/46 (100%)	100	98–100
*flgS*	46/46 (100%)	100	98–100
*flhA*	46/46 (100%)	100	98–100
*flhB*	46/46 (100%)	100	98–100
*flhF*	46/46 (100%)	100	99–100
*flhG*	46/46 (100%)	100	99–100
*fliA*	46/46 (100%)	100	98–100
*fliD*	46/46 (100%)	19–100	96–100
*fliE*	46/46 (100%)	100	99–100
*fliF*	46/46 (100%)	100	98–100
*fliG*	46/46 (100%)	100	99–100
*fliH*	46/46 (100%)	100	98–100
*fliI*	46/46 (100%)	100	97–100
*fliK*	46/46 (100%)	97–100	94–100
*fliL*	46/46 (100%)	100	97–100
*fliM*	46/46 (100%)	33–100	98–100
*fliN*	46/46 (100%)	100	98–100
*fliP*	46/46 (100%)	100	99–100
*fliQ*	46/46 (100%)	100	98–100
*fliR*	46/46 (100%)	100	98–100
*fliS*	46/46 (100%)	100	99–100
*fliW*	46/46 (100%)	100	100
*fliY*	46/46 (100%)	100	99–100
*maf4*	38/46 (83%)	4–100	83–100
*motA*	46/46 (100%)	100	94–100
*motB*	46/46 (100%)	100	94–100
*pflA*	46/46 (100%)	100	98–100
*pseA*	46/46 (100%)	100	96–100
*pseB*	46/46 (100%)	100	89–100
*pseC*	46/46 (100%)	100	92–100
*pseD/maf2*	39/46 (85%)	5–100	76–99
*pseE/maf5*	39/46 (85%)	14–100	79–100
*pseF*	46/46 (100%)	95–100	94–100
*pseG*	46/46 (100%)	98–100	87–100
*pseH*	46/46 (100%)	87–100	84–94
*pseI*	46/46 (100%)	99–100	93–98
*ptmA*	39/46 (85%)	47–100	94–100
*ptmB*	39/46 (85%)	45–100	96–100
Invasion			
*rpoN*	46/46 (100%)	100	98–100
*ciaB*	46/46 (100%)	100	98–100
*ciaC*	46/46 (100%)	100	96–100
*sinH*	1/46 (2%)	10	100
*ssaL*	1/46 (2%)	26	99
*invA*	1/46 (2%)	7	100
*invG*	1/46 (2%)	13	100
Toxin			
*cdtA*	46/46 (100%)	100	98–100
*cdtB*	46/46 (100%)	93–100	96–100
*cdtC*	46/46 (100%)	100	98–100
Chemotaxis			
*cheA*	46/46 (100%)	100	98–100
*cheV*	46/46 (100%)	100	99–100
*cheW*	46/46 (100%)	100	99–100
*cheY*	46/46 (100%)	100	99–100
*cheV3*	46/46 (100%)	13	76–77
*tlpA*	34/46 (74%)	7–8	77–82
*tlpB*	9/46 (20%)	9	77
*tlpC*	6/46 (13%)	8–11	76–78
T6SS/pVir plasmid			
*Cjp54*	2/46 (4%)	100	95–100
*virB4*	2/46 (4%)	49–100	91–100
*virB8*	2/46 (4%)	100	95–100
*virB9*	2/46 (4%)	64–100	92–98
*virB10*	2/46 (4%)	80–100	96
*virB11*	2/46 (4%)	99	94–99
*virD4*	2/46 (4%)	53–100	99–100
Metabolism			
* cysC*	43/46 (93%)	100	98–100

The additional 20 virulence-related genes searched were detected in all 46 *C. jejuni* strains studied. The similarity rate varied between 96 and 100% of identity for all strains with coverage between 98 and 100% (Table 3). No significant correlation was observed between the origin of the *C. jejuni* strains studied and the expression of the evaluated virulence genes.

**Table 3:** Characteristics of the 20 virulence related genes searched in 46 *Campylobacter jejuni* strains studied

Gene	Query cover (%)	Identity (%)	Product function	Related to stress
*katA*	98 −100	96–100	catalase	acid and oxidative
*perR*	100	98–99	peroxide stress regulator	acid and oxidative
*fur*	100	98–100	fur ferric uptake regulator	oxidative
*csrA*	100	99–100	carbon storage regulator	oxidative
*sodB*	100	99–100	superoxide dismutase (Fe)	acid and oxidative
*ahpC*	100	98–100	alkyl hydroperoxide reductase	acid and oxidative
*ccpA-2*	100	97–99	Cytochrome c551 peroxidase	oxidative
*tpx*	100	99–100	2-Cys peroxiredoxin	
*htrA*	100	97–100	serine protease (protease DO)	acid, osmotic and oxidative
*fdxA*	100	96–100	ferredoxin	oxidative
*dcuA*	100	96–100	anaerobic C4-dicarboxylate transporter	oxidative
*grpE*	99–100	93–100	heat shock protein	acid, osmotic and oxidative
*hspR*	100	98–100	heat shock transcriptional regulator	acid and osmotic
*dnaK*	100	96–100	Chaperone protein	acid, osmotic and oxidative
*spoT*	100	98–100	putative guanosine-3',5'-bis(diphosphate) 3'- pyrophosphohydrolase	oxidative
*argF*	100	97–100	delta-aminolevulinic acid dehydratase	acid, cold and oxidative
*asd*	100	98–100	aspartate-semialdehyde dehydrogenase	cold and oxidative
*proS*	100	97–99	proline–tRNA ligase	acid and cold
*hupB*	100	99–100	NA-binding protein	cold and oxidative
*rpoB*	100	98–100	DNA-directed RNA polymerase subunit beta	acid, cold and oxidative

## Discussion

*Campylobacter jejuni* has been a major foodborne pathogen that causes gastroenteritis in many countries [2, 4, 5]. In Brazil, campylobacteriosis remains underdiagnosed and underreported, and studies on this pathogen are still limited [9–13, 16]. This study assessed the survival of *C. jejuni* strains isolated from humans, animals, and food, between 1996 and 2016 in Brazil by assessing survival under different stress conditions, invasion and survival in Caco-2 cells and U-937 macrophages, virulence in *G. mellonella,* and the frequency of virulence related genes.

In the present study, all *C. jejuni* strains studied showed growth at 4 °C and 37 °C. After 24 h at 4 °C, 35 (76%) strains survived greater than the reference strain *C. jejuni* ATCC 33291 (Fig. 1b), while at 37 C, 41 (89%) strains showed similar survival after 24 h of incubation (Fig. 1d). Previous studies have also reported that *C. jejuni* survived better at refrigeration temperatures (4–7 °C) than at higher temperatures (20–30 °C) [34–36].

These results are an alert and may suggest that *C. jejuni* strains can survive during storage at refrigerated temperature (4° C) of poultry meat products becoming a risk to the consumer, since ingestion of 500 to 800 *C. jejuni* cells may cause gastroenteritis [36, 37]. In addition, surviving at 37 °C is also an advantage to the bacteria as this is the temperature of the human body.

As a foodborne pathogen, *C. jejuni* must pass through hostile environments in the digestive tract before colonizing the intestine. To maintain pH homeostasis under acidic conditions, the bacterium activates mechanisms that limit H⁺ entry, promote its efflux, and repair acid-induced cellular damage [19, 20]. In this study, 40 (87%) strains showed survival equal to or greater than the reference strain *C. jejuni* ATCC 33291 after two hours of incubation in BHI at pH 4.5 (Fig. 2c).

Survival under acidic conditions is essential for *C. jejuni* to pass through the stomach and establish infection, where it is also exposed to other stresses such as reactive oxygen and nitrogen species [19, 38]. The ability to tolerate acid stress directly affects the infective dose of enteric pathogens. In this way, the low infective dose of 500 to 800 organisms for *C. jejuni* [37] suggests that this bacteria is well equipped to sense and respond in an environment with a sudden drop in pH.

*C. jejuni* is more sensitive to acid exposure than other foodborne pathogens such as *Salmonella*, *E. coli,* and *Helicobacter pylori* [39, 40]. This sensitivity has been associated with the absence of stress response regulators including RpoS, SoxRS, and OxyR, as well as osmotic shock protectants such as BetAB [41]. However, *C. jejuni* does contain the global ferric uptake regulator (Fur) that regulates genes in response to iron transport, metabolism, and oxidative stress defense [24, 42].

The survival of *Campylobacter* under oxidative stress is influenced by its microaerophilic nature [18, 43]. Although some studies suggested that these bacteria can adapt to aerobic conditions [43], in the present study only 16 (35%) of the 46 *C. jejuni* strains showed growth after incubation at 42 °C under aerophilic conditions for 24 h. After exposure to oxidative stress, only 10 strains survived after 10 min of incubation (Fig. 3a), and two strains (CCAMP 1025 and CCAMP 1523), both isolated from food, remained viable after 1 h. This limited survival was expected given the microaerophilic nature of *C. jejuni*.

Sodium chloride (NaCl) is widely used as a food preservative and microbial inhibitor. Although, *C. jejuni* typically grows in the presence of 0.5–1.5% NaCl and higher concentrations may reduce viability [44], all strains in this study remained viable after two hours of incubation in BHI containing 7.5% NaCl. Notably, 39 (85%) strains showed greater survival than the reference strain *C. jejuni* ATCC 33291 (Fig. 3c). This ability to tolerate osmotic stress may favor the persistence and transmission of the pathogen in food environments where high solute concentrations have been used for microbial control [44].

According to the results obtained in the present work, the behavior of *C. jejuni* strains studied under different stress conditions is probably strain-dependent and is not related to the year, place, or source of isolation.

The presence of the virulence-related genes *katA*, *perR*, *fur*, *csrA*, *sodB*, *ahpC*, *ccpA-2*, *tpx*, *htrA*, *fdxA*, *dcuA*, *grpE*, *hspR*, *dnaK*, *spoT*, *argF*, *asd*, *proS*, *hupB* and *rpoB* were analyzed using WGS data of the 46 *C. jejuni* strains using BLASTn and all the strains studied presented all the genes searched (Table 3). It is important to point out that not only the presence but also the expression level of those genes is essential to promote *C. jejuni* survival under adverse conditions which has been demonstrated by molecular techniques such as RT-qPCR, microarray and/or transcriptome [21, 22, 44, 45].

*C. jejuni* contains several genes encoding important oxidative stress response proteins. Catalase (KatA) converts H₂O₂ into H₂O and O₂, while superoxide dismutase (SodB) degrades superoxide radicals. Other proteins such as AhpC, FdxA, HtrA, and HspR also contribute to aerobic stress tolerance [21, 22, 44]. In addition, regulatory proteins including Fur, PerR, and CsrA, as well as stress-related proteins such as Tpx, GrpE, and DnaK, play important roles in oxidative stress defense and adaptation to adverse conditions [21, 23, 25, 26, 44, 45].

Several cellular models have been used to evaluate the ability of *C. jejuni* to invade the gastrointestinal tract, with Caco-2 epithelial cells being one of the most commonly used to mimic the human intestinal environment [46, 47]. In this study, all 46 *C. jejuni* strains demonstrated the ability to invade Caco-2 cells compared to the reference strain ATCC 33291 (Fig. 4a).

Similar findings were reported by [48], where most (70%) *C. jejuni* strains isolated from different sources showed higher invasion capacity than the control strain *C. jejuni* NCTC 81116. In contrast, another study evaluating 57 human isolates in China reported that 68% of the strains exhibited low invasion capacity compared to the same control strain [49].

Likewise, for the survival assay in U-937 macrophages, all 46 *C. jejuni* strains studied showed the ability to survive in these cells, with 36 strains invading at levels higher than the reference strain ATCC 33291 (Fig. 4b). These findings, together with the invasion results observed in Caco-2 epithelial cells, reinforced the pathogenic potential of the studied strains and their ability to evade host immune defenses.

*Galleria mellonella* larvae represent a convenient infection model due to their low cost, ease of cultivation, and innate immune components, such as hemocytes and opsonins, which are similar to those of mammals. However, this model lacks the adaptive immune response found in vertebrates [50].

In the present study, 23 representative *C. jejuni* strains were selected for evaluation in the *G. mellonella* infection model based on their phenotypic performance in the *in vitro* assays. Therefore, the results should be interpreted considering that only a subset of the strains was analyzed. This virulence assay grouped the strains according to their virulence profiles (Fig. 5a–c), and it is worth noting that the distribution of strains by virulence groups was very similar between the different isolation sources. Notably, 64% of the strains were classified as virulent or intermediate, showing virulence equal to or greater than the reference strain *C. jejuni* ATCC 33291.

Previous studies in the United Kingdom have also reported strain-dependent differences in the survival of *G. mellonella* larvae infected with *C. jejuni*, with larval survival ranging from 70–90% [51], 50–70% [31], 50–90% [52], and 0–90% [53].

The analysis of virulence genes in the 46 *C. jejuni* genomes using ABRicate and the VFDB database showed a very similar gene distribution among the strains. Several genes essential for *C. jejuni* pathogenesis were identified with high coverage and identity, reinforcing the pathogenic potential of the studied strains, as invasion and survival in host cells are closely associated with the presence and expression of virulence genes [17].

Panzenhagen and colleagues [54] analyzed 40,371 *C. jejuni* genomes from multiple continents and identified a virulome composed of 126 virulence genes among 2,597 total genes. Many results corroborate with the result of the present work, including the high frequency of key virulence genes such as *cadF*, *ciaB*, *virB*, and *wlaN*. However, differences were observed in the frequencies of *flaA* and *flaB*, which are related to flagellum formation and motility.

Most of the 139 genes listed in Table 2 and all 20 genes in Table 3 were detected at frequencies above 90% among the 46 *C. jejuni* strains analyzed. Notably, 89 (64%) of the genes in Table 2 were present at very high frequencies, and nearly all genes involved in the motility apparatus were detected in 100% of the strains (Table 2). These findings indicated that the strains isolated from different sources in Brazil harbor a broad and conserved virulome, including genes associated with colonization, biofilm formation, motility/invasion, toxin production, and chemotaxis.

In agreement with previous studies [54, 55], the plasmid-associated *virB-D* genes (pVir) were detected at low frequency (4%) in the present study. Similarly, Panzenhagen et al. [54] reported a prevalence below 5% for these genes in *C. jejuni* genomes from different regions worldwide, suggesting that the T4SS encoded by the pVir plasmid may represent part of the accessory genome rather than a major determinant of virulence.

The *wlaN* gene showed a moderate prevalence, being detected in 24 (52%) of the 46 strains analyzed (Table 2). Comparable frequencies were reported by Panzenhagen et al. [54], who observed a prevalence of 56% in *C. jejuni* genomes from multiple continents. However, lower frequencies have been described in isolates from Japan [56], Ireland [30], and Brazil [14]. The *wlaN* gene encodes a β−1,3-galactosyltransferase involved in cell wall synthesis and has been associated with molecular mimicry of neuronal gangliosides, which may contribute to the development of Guillain–Barré syndrome following *C. jejuni* infection [57]. Despite its potential role in virulence, the variable prevalence of *wlaN* suggests that its contribution to *C. jejuni* pathogenicity requires further investigation.

In contrast to the findings of [54], who reported a prevalence below 35% for the *flaA* and *flaB* genes, both flagellin-encoding genes were detected in 100% of the 46 *C. jejuni* strains analyzed in the present study, with high coverage and identity (Table 2). Similar discrepancies have been reported in other studies; for example, [58] identified *flaA* and *flaB* in only 11.11% of 81 *Campylobacter* strains isolated in Chile. These differences are notable, particularly because both studies used the Virulence Factors Database (VFDB) to determine the virulome. Interestingly, in [54], *flaA* and *flaB* were the only genes related to motility and flagellum formation detected at low frequency among thousands of genomes analyzed.

Overall, the presence of virulence-related genes in the *C. jejuni* strains highlights their potential role in stress response and bacterial survival. Nevertheless, further studies are needed to better understand the mechanisms underlying survival under different stress conditions and their relationship with virulence genes, particularly at the transcriptional level.

## Conclusions

In conclusion, the pathogenic potential of the *C. jejuni* strains studied was highlighted by the high rates of survival under different stress conditions, by the invasion and survival in Caco-2 cells and U-937 macrophages, the virulence in *Galleria mellonella* and by the presence of some important virulence-related genes in those strains. The results obtained in the present study allowed a better understanding of the *C. jejuni* strains isolated in the country and suggested that more rigorous control measures of this pathogen in food, especially poultry products, may be needed in Brazil, an important producer and exporter of chicken meat in the world.

## Supplementary Information

Below is the link to the electronic supplementary material.

Supplementary file1 (PDF 94 KB)

## Data Availability

The datasets generated and/or analysed during the current study are available in the GenBank repository [https://www.ncbi.nlm.nih.gov/bioproject/PRJNA258022].
