# Supplementary material for: Campylobacter jejuni strains isolated in Brazil have important virulence related genes and survive to different stress conditions
Source: Braz J Microbiol. 2026 Apr 1;57(1):102. doi: 10.1007/s42770-026-01928-6 (PMC13043972; doi:10.1007/s42770-026-01928-6)
Supplement: Supplementary file 1 — Supplementary file1 (PDF 94 KB) [file 42770_2026_1928_MOESM1_ESM.pdf]

(S1)

Grouping information using Tukey method and 95% confidence

Factor	N	Averages	Group				
CCAMP 487	3	5,350	A				
CCAMP 685	3	4,594	A	B			
CCAMP 699	3	4,106		B	C		
CCAMP 1497	3	3,417			C		
CCAMP 1518	3	3,361			C		
CCAMP 1523	3	1,556				D	
CCAMP 1025	3	0,8278				D	E
Cj 07	3	0,1689					E
CCAMP 601	3	0,05648					E
Cj 01	3	0,002944					E

Averages that do not share the same letter are significantly different.

Figure S1 – Bidirectional analysis of variance (ANOVA). Different letters indicate statistically significant differences between means at a 5% significance level.
